# Esophageal tortuosity in achalasia: increased length-to-height ratio predicts inferior symptom relief and esophageal emptying following myotomy

**DOI:** 10.1007/s00464-024-11200-3

**Published:** 2024-10-14

**Authors:** John O. Barron, Nethra Jain, Andrew J. Toth, Soon Moon, Eugene H. Blackstone, Sadia Tasnim, Madhusudhan Sanaka, Monisha Sudarshan, Mark E. Baker, Sudish C. Murthy, Siva Raja

**Affiliations:** 1 Department of Thoracic and Cardiovascular Surgery, Heart, Vascular, and Thoracic Institute, Cleveland Clinic, 9500 Euclid Avenue/Desk J4-133, Cleveland, OH 44915 USA; 2 Department of Quantitative Health Sciences, Research Institute, Cleveland Clinic, Cleveland, OH USA; 3 Department of General Surgery, South Pointe Hospital, Cleveland Clinic, Cleveland, OH USA; 4 Department of Gastroenterology and Hepatology, Digestive Disease and Surgery Institute, Cleveland Clinic, Cleveland, OH USA; 5 Digestive Disease and Surgery Institute and Imaging Institute, Cleveland Clinic, Cleveland, OH USA

**Keywords:** Esophageal achalasia, Morphology, Tortuosity, Length-to-height ratio, Outcomes, Manometry

## Abstract

**Background:**

Current classification of achalasia does not account for variability in esophageal tortuosity. The esophageal length-to-height ratio (LHR) was developed to objectively quantify tortuosity, based on the premise that the esophagus must elongate to become tortuous. Hence, we assess the relationship of esophageal tortuosity, measured by LHR, to preoperative patient characteristics and post-myotomy outcomes, including longitudinal symptom relief and esophageal emptying.

**Methods:**

From 01/2014 to 01/2020, 420 eligible adult patients underwent myotomy for achalasia at our institution, 216 (51%) Heller myotomy and 204 (49%) per-oral endoscopic myotomy. LHR was measured on pre- and first postoperative timed barium esophagram (TBE), with larger values signifying greater tortuosity. Variable predictiveness and risk-adjusted longitudinal estimates of symptom relief (Eckardt score ≤ 3) and complete emptying, in relation to LHR and manometric subtype, were estimated.

**Results:**

Median [15th, 85th percentile] preoperative LHR was 1.04 [1.01, 1.10]. Preoperative esophageal width > 3 cm and age > 68 years were most predictive of increased LHR. Increased LHR corresponded with decreases in longitudinal postoperative symptom relief and complete esophageal emptying, with a 4% difference in symptom relief and 20% difference in complete emptying, as LHR increased from 1.0 to 1.16. After adjusting for patient factors, including LHR, manometric subtype was less predictive of symptom relief, with estimated symptom relief occurring in 4% fewer patients with Type III achalasia, compared to Types I and II. Overall, LHR decreased following myotomy in patients with an initially tortuous esophagus.

**Conclusion:**

Length-to-height ratio was the only variable highly predictive of both longitudinal post-myotomy symptom relief and complete esophageal emptying, whereas manometric subtype was less predictive. These findings highlight the importance of tortuosity in the treatment of patients with achalasia, suggesting that inclusion of esophageal morphology in future iterations of achalasia classification is warranted.

**Graphical abstract:**

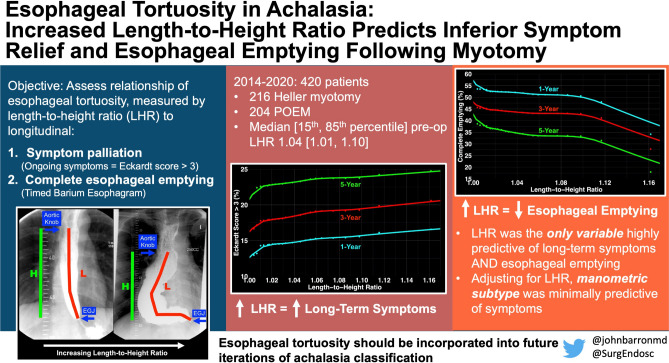

**Supplementary Information:**

The online version contains supplementary material available at 10.1007/s00464-024-11200-3.

In patients with achalasia, a non-relaxing lower esophageal sphincter coupled with ongoing obstruction can lead to a dilated and tortuous esophagus and eventual “sigmoidal” morphology [1]. Thus far, classification of esophageal achalasia has focused on manometric criteria; however, there are often substantial differences in esophageal morphology between patients, with a massively dilated and tortuous esophagus thought to be associated with inferior outcomes [2–4]. That said, esophageal tortuosity is a continuum, the severity of which our previous work has shown to be subjective and variable, even among experts [5]. For this reason, we developed the esophageal length-to-height ratio (LHR), an objective method to quantify esophageal tortuosity, based on the premise that the esophagus must elongate to become tortuous [5]. Hence, we assess the relationship of esophageal tortuosity, measured by length-to-height ratio, to preoperative patient characteristics and postoperative clinical outcomes, including longitudinal symptom relief and esophageal emptying.

## Patients and methods

### Study population

From January 2014 to January 2020, 561 adult patients underwent surgical myotomy (Heller myotomy or per-oral endoscopic myotomy (POEM)) for achalasia, 141 of whom were excluded due to a previous history of Heller myotomy or lack of preoperative timed barium esophagram (TBE) for LHR measurement. Treatment decisions were made according to our previously published treatment algorithm for esophageal achalasia at the Cleveland Clinic [6]. Of the remaining 420 patients, one-hundred ninety-nine (47%) were female, with a median age of 56 years (Table 1). Preoperative high-resolution manometry demonstrated 71 (19%) patients with Type I, 234 (63%) with Type II, 42 (11%) with Type III achalasia, and 27 (7%) unspecified. Two-hundred sixteen (51%) underwent Heller myotomy and 204 (49%) POEM, with Dor fundoplication performed in 203 (94%) of the patients that underwent Heller myotomy.

**Table 1:** Baseline and operative characteristics of patients undergoing surgical myotomy for achalasia

Variable	*N*	Count (%) or 15/50/85 percentiles
Age	420	36/56/74
Female	420	199 (47)
Race	420	
White		364 (87)
Black		34 (8)
Asian		6 (1.4)
Hispanic		6 (1.4)
Other		10 (2.4)
ASA^a^ class	420	
I		4 (0.97)
II		74 (18)
III		224 (54)
IV		110 (26)
Body mass index	412	21.6/26.9/34.2
Achalasia type		
I		71 (19)
II		234 (63)
III		42 (11)
Unknown		27 (7)
Integrated relaxation pressure	384	5.58/22.1/36.3
Prior interventions		
Botulinum toxin	412	83 (20)
Pneumatic dilation		37 (9)
Simple dilation		76 (18)
Pre-Op Eckardt Score	339	4/7/9
0		2 (0.6)
1		4 (1.2)
2		14 (4.1)
3		14 (4.1)
4		30 (8.8)
5		34 (10)
6		57 (17)
7		48 (14)
8		44 (13)
9		50 (15)
10		23 (6.8)
11		11 (3.2)
12		8 (2.4)
Barium ingested, mL	406	100/235/250
TBE^b^ width, cm	407	
1 min		1.7/3/4.7
5 min		0.1/2.5/4.4
TBE height, cm	407	
1 min		5.5/10.5/17
5 min		0.5/9/15.5
Length-to-height ratio	420	1.01/1.04/1.10
Diverticulum	420	22 (5)
Hiatal hernia	420	29 (7)
Myotomy type	420	
Heller myotomy		216 (51)
POEM		204 (49)
Surgical approach	216	
Robotic		110 (51)
Laparoscopic		92 (43)
Open		14 (7)
Fundoplication type	216	
None		1 (0.5)
Dor		203 (94)
Toupet		11 (5)

^a^American Society of Anesthesiologists, ^b^Timed Barium esophagram

### Surgical technique

Heller myotomy was routinely performed laparoscopically or robotically, usually with Dor fundoplication, as described by Fezcko and colleagues [7]. Unless there was a hiatal hernia, only the anterior esophagus was mobilized. A full-thickness anterior myotomy was performed over a 60F Maloney bougie along 4–6 cm of the distal esophagus and 3 cm of the stomach. For POEM procedures, a 6–7 esophageal myotomy (8–9 cm for type III achalasia) was performed, extending 2–4 cm further onto the gastric wall. The mucosotomy was then closed with endoscopic clips.

### Esophageal length-to-height ratio

**Fig. 1:**
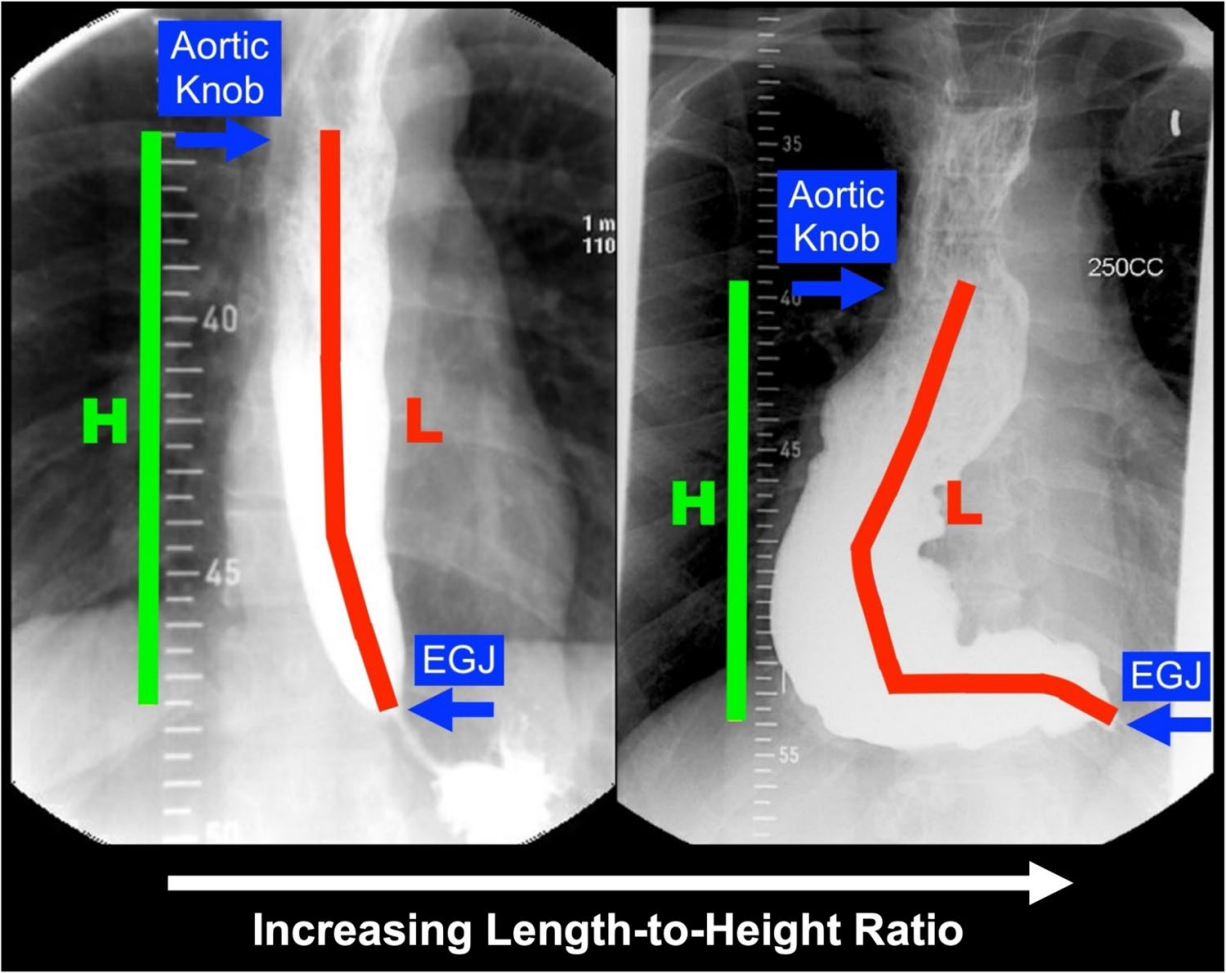
Measurement of esophageal length (red) and height (green) on timed barium esophagrams with increasing severity of tortuosity. Division of length by height results in the length-to-height ratio

Esophageal length and height were measured by two study personnel via image viewing software within the electronic medical record, as previously described [5]. The preoperative length-to-height ratio was measured on the 1-min TBE radiograph obtained at the preoperative visit, typically 1–3 months prior to myotomy. The esophageal “length” represented a line drawn along the mid lumen of the esophagus, from the inferior aspect of the aortic knob, until the esophagogastric junction, highlighted by the end of the “bird’s beak” (Fig. 1). A separate vertically oriented “height” line was then drawn, directly between the same landmarks. Division of the “length” by the “height” generated the length-to-height ratio. Hence, patients with a completely straight esophagus had a length to height ratio of 1.0, with larger length-to-height ratios signifying increased esophageal tortuosity. Postoperative length-to-height ratio was measured on the 1-min TBE radiograph performed at the 3-month follow-up visit.

### Data

The Cleveland Clinic Institutional Review Board approved this cohort study on September 9, 2023 (Protocol 23-912), with waiver of patient consent. Baseline, procedural, and postprocedural data were obtained from the electronic medical record, maintained in a Research Electronic Data Capture (REDCap) Achalasia Longitudinal Database, and requested in deidentified form in compliance with Health Insurance Portability and Accountability Act standards. Standard definitions of variables, including demographics, comorbidities, treatment(s), and outcomes, were used.

### Follow-up

Patients underwent both pre- and postoperative assessment with timed barium esophagram (TBE) and Eckardt score. Following myotomy, patients were evaluated at 2 weeks, 3 months, and annually for 3 years. If symptoms (Eckardt ≤ 3) and emptying remained stable at 3 years, they were subsequently evaluated every 2–3 years, or sooner if symptoms recurred. Patients underwent TBE and assessment of symptoms via Eckardt score at each visit [8, 9]. Follow-up was performed during in-person office visits or, if patients did not follow-up in person, symptoms were assessed via telephone call (Supplemental Fig. 1). From 1 to 3 years postoperatively, 367 TBEs were performed in 245 patients and 420 Eckardt scores collected in 256 patients (Supplemental Fig. 2).

### Endpoints

Endpoints were (1) baseline patient characteristics predictive of preoperative length-to-height ratio, variable predictiveness of (2) postoperative longitudinal symptom relief measured by Eckardt score and (3) complete esophageal emptying on TBE prior to any reintervention(s), and (4) post-operative change in length-to-height ratio. Inadequate symptom relief was defined by Eckardt score >3 and complete TBE emptying defined as TBE column height and width of 0 cm at 5 min [9].

### Statistical analyses

Analyses were performed using SAS version 9.4 (SAS Institute, Inc) and R 4.2.1 software (R Foundation for Statistical Computing). Continuous variables are summarized as median [15th, 85th percentiles]. Categorical data are summarized as frequencies and percentages.

#### Preoperative characteristics and length-to-height ratio

Random forest machine learning methodology was used to assess the association of preoperative patient characteristics with length-to-height ratio, with 5000 trees created for each forest using the variables in Supplemental Table 1 [10]. Eleven variables were used for each split, with missing data imputed on the fly [11]. Random forest methods were used to calculate variable importance (VIMP) in predicting length-to-height ratio [12, 13]. Partial dependency plots were used to visualize the relationships between patient characteristics and LHR [12, 13]. A more detailed description of the analytic approach, including Random Forest methods and VIMP, is provided as Appendix 1.

#### Symptom relief, esophageal emptying, and postoperative length-to-height ratio

We assessed relationships of preoperative LHR and other baseline patient characteristics with longitudinal symptom relief and complete emptying using boostmtree, a boosting approach based on a marginal model for modeling longitudinal data that adjusts for all included features (Supplemental Table 1) [14]. Variables predictive of inadequate symptom relief (Eckardt score >3) and complete 5-min postoperative esophageal emptying were selected using variable importance, with partial dependence plots generated to visualize these relationships [12, 13]. Postoperative length-to-height ratio, measured at 3-month follow-up, was plotted in reference to preoperative length-to-height ratio to understand the effect of myotomy on severity of tortuosity.

## Results

### Patient and operative characteristics

#### Preoperative length-to-height ratio

**Fig. 2:**
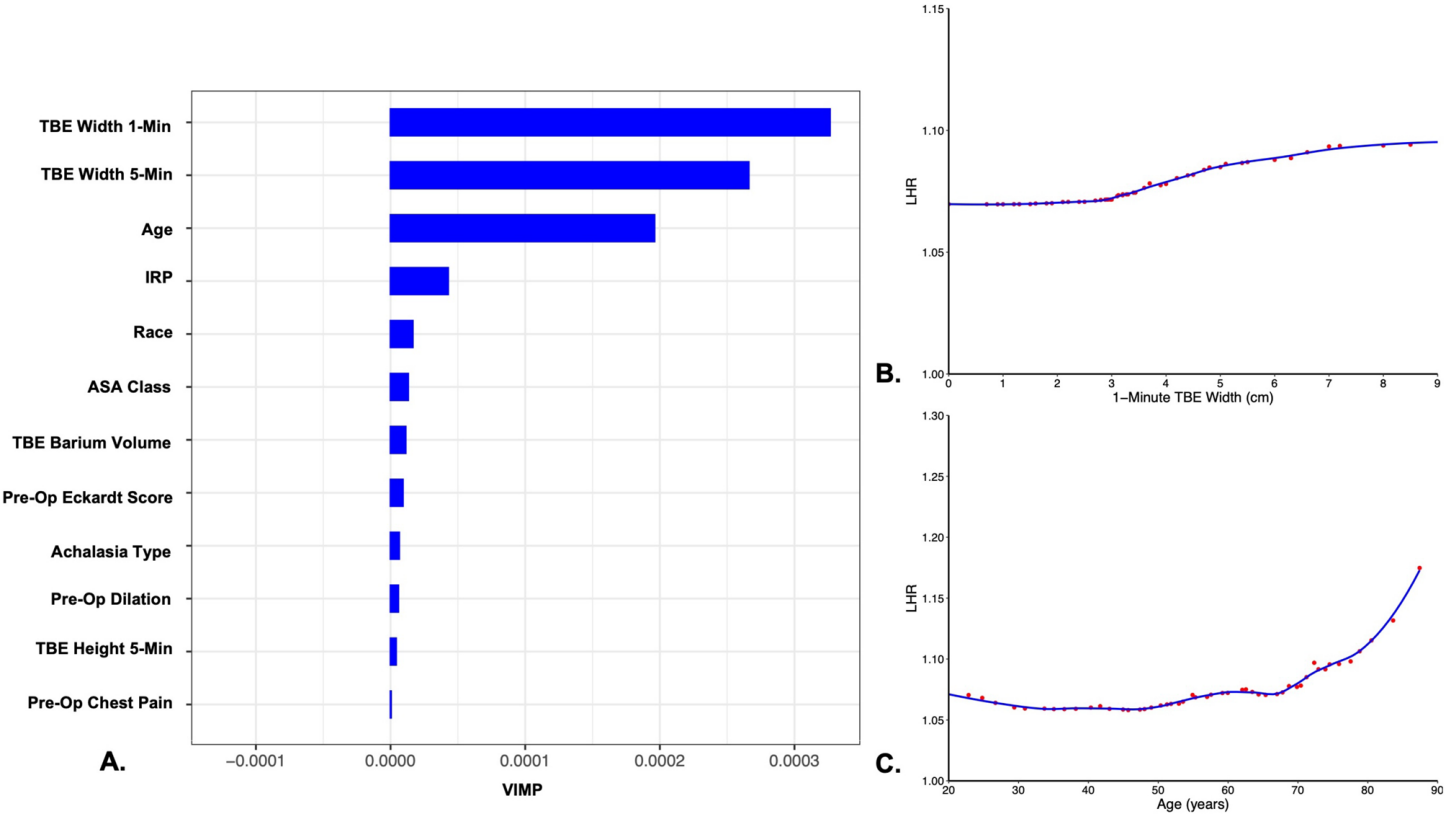
Preoperative variables predictive of preoperative length-to-height ratio. **A** Variables of maximal importance and partial dependency plots based on **B** 1-min TBE width and **C** patient age. *TBE* timed barium esophagram, *IRP* integrated relaxation pressure, *ASA* American Society of Anesthesiologist, *VIMP* variable of maximal importance

Preoperative length-to-height ratios ranged from 1.0 to 1.77, with a median of 1.04 [1.01, 1.10]. Esophageal width and patient age were the baseline patient characteristics most predictive of length-to-height ratio (Fig. 2A). Preoperative 1-min TBE widths greater than about 3 cm and ages greater than about 68 years were associated with an increase in LHR, suggesting greater tortuosity (Fig. 2B, C). Other factors, including achalasia subtype, preoperative symptom severity, sex, and body mass index were minimally predictive of preoperative LHR.

### Symptom relief

**Fig. 3:**
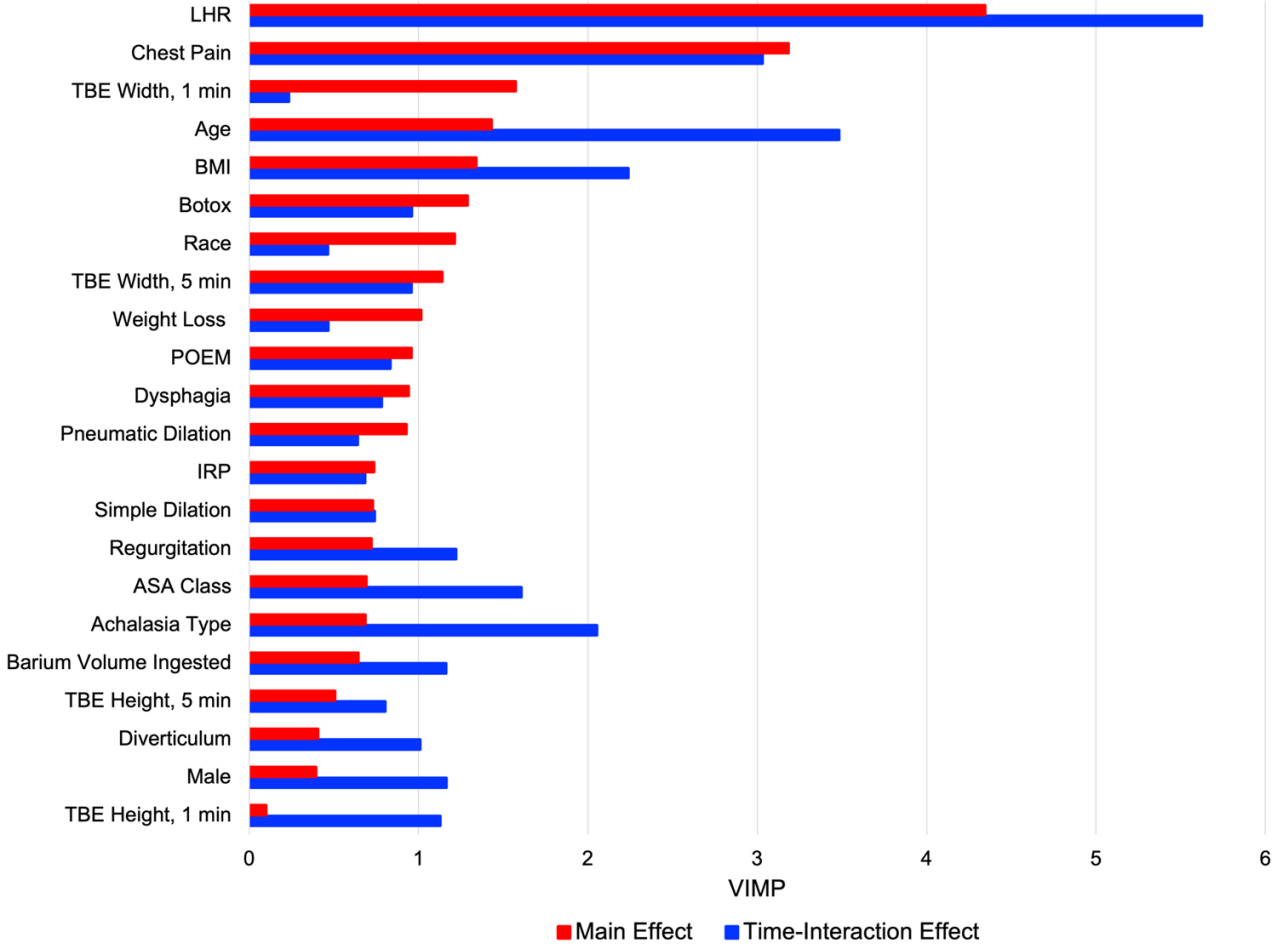
Preoperative variable importance in predicting postoperative incomplete symptom relief, defined as Eckardt score >3. The main effect is independent of time, while the time-interaction effect changes with time. *TBE* timed barium esophagram, *LHR* length-to-height ratio, *ASA* American Society of Anesthesiologists, *POEM* per-oral endoscopic myotomy, *BMI* body mass index, *IRP* integrated relaxation pressure, *ASA* American Society of Anesthesiologists, *VIMP* variable of maximal importance

**Fig. 4:**
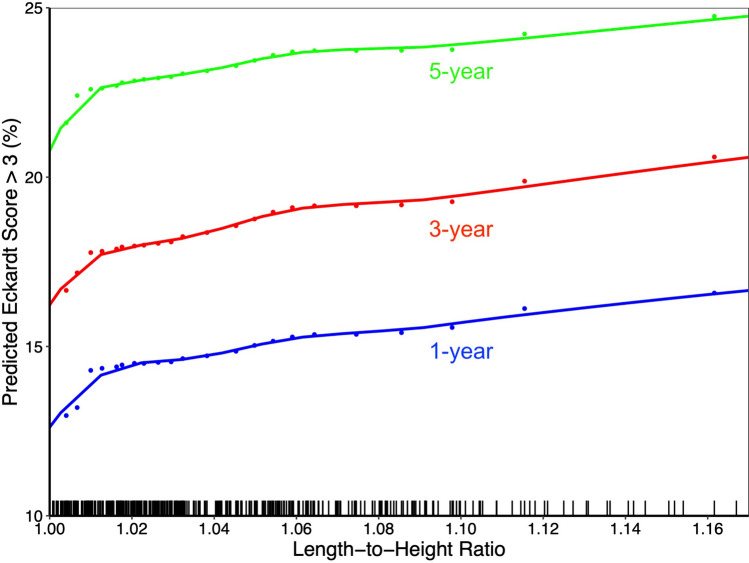
Estimated longitudinal postoperative incomplete symptom relief, defined by Eckardt score >3, at 1, 3, and 5 years following myotomy. Rug marks represent individual patient length-to-height ratio measurements

Length-to-height ratio was the variable most predictive of incomplete longitudinal symptom relief, defined as Eckardt score >3, with manometric subtype of less predictive value (Fig. 3). An increase in the preoperative LHR corresponded with a gradual progressive increase in the percentage of patients with incomplete symptom relief 1, 3, and 5 years postoperatively, according to risk-adjusted estimates (Fig. 4). This corresponded to about 21% incomplete symptom relief with LHR of 1.0 at 5 years, compared to about 25% with LHR of 1.16 (Fig. 4).

**Fig. 5:**
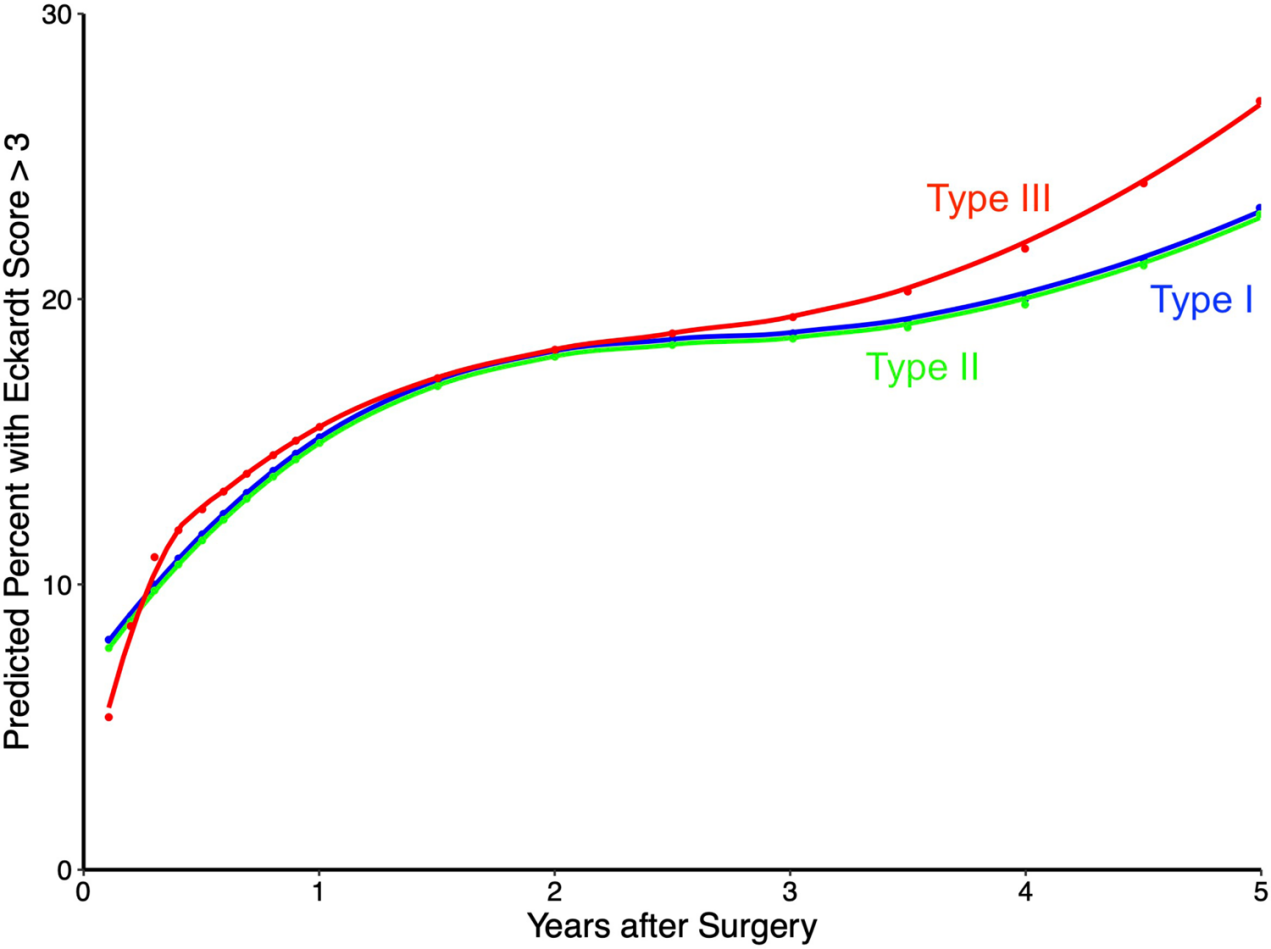
Estimated longitudinal postoperative incomplete symptom relief, defined by Eckardt score >3, according to achalasia manometric subtype

Adjusting for preoperative patient factors, including LHR and esophageal width, estimated symptom relief was comparable among all three achalasia subtypes until 3 years. After 3 years, patients with type III achalasia were more likely to have incomplete symptom relief, with about 27% incomplete symptom relief at 5 years with Type III achalasia, vs about 23% with Types I and II achalasia (Fig. 5).

### Complete esophageal emptying

**Fig. 6:**
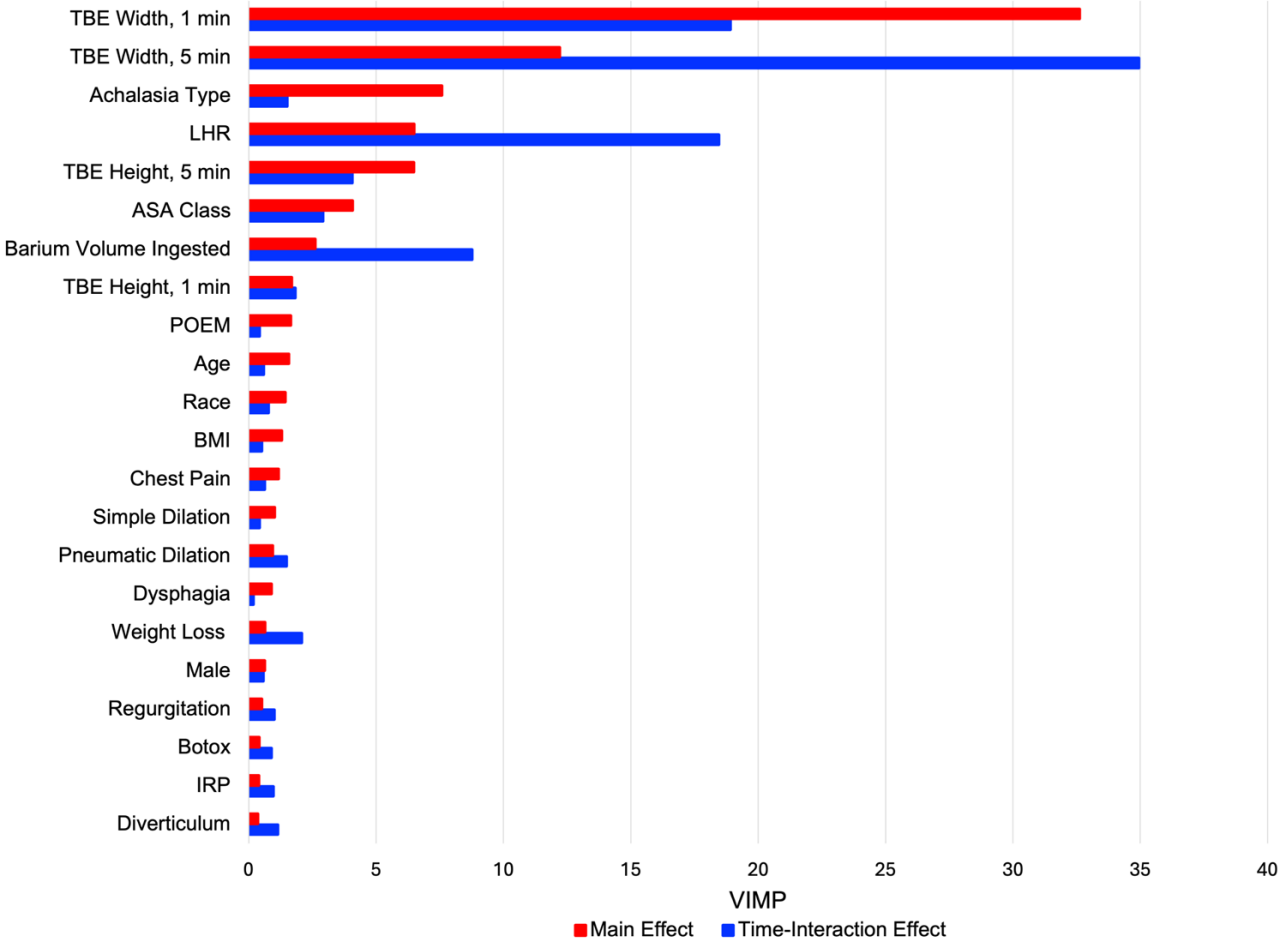
Preoperative variable importance in predicting longitudinal postoperative complete esophageal emptying within 5-min. The main effect is independent of time, while the time-interaction effect changes with time. *TBE* timed barium esophagram, *LHR* length-to-height ratio, *ASA* American Society of Anesthesiologists, *POEM* per-oral endoscopic myotomy, *BMI* body mass index, *IRP* integrated relaxation pressure, *ASA* American Society of Anesthesiologists, *VIMP* variable of maximal importance

**Fig. 7:**
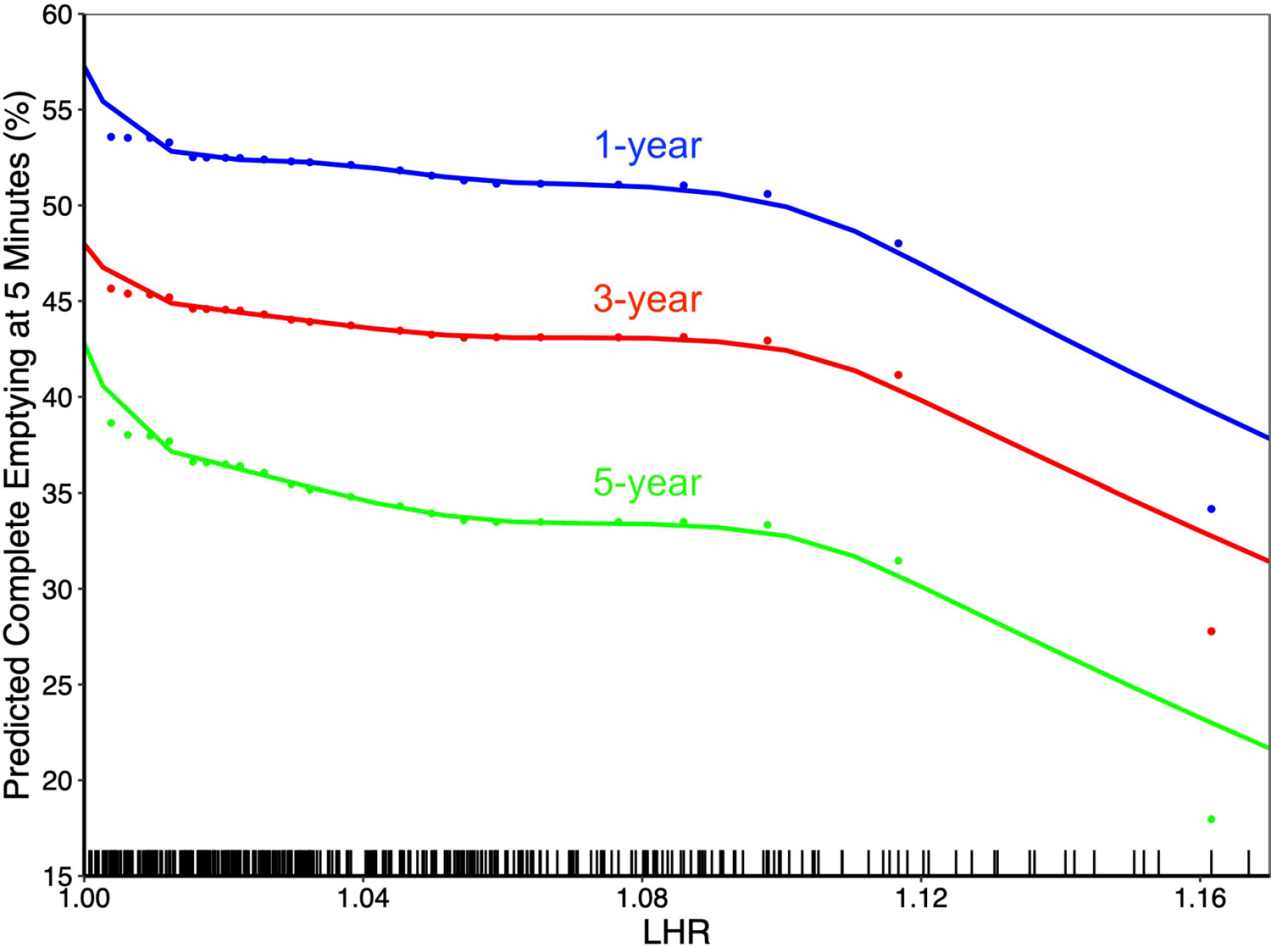
Estimated 5-min complete esophageal emptying according to preoperative length-to-height ratio at 1, 3, and 5 years following myotomy. Rug marks represent individual patient length-to-height ratio measurements

Preoperative esophageal width, manometric subtype, and LHR were the variables most predictive of longitudinal postoperative complete esophageal emptying, with predictiveness varying over time (Fig. 6). An increase in the preoperative LHR corresponded to a progressive decrease in complete esophageal emptying 1, 3, and 5 years postoperatively, with risk-adjusted estimates of about 43% complete emptying with LHR of 1.0 at 5 years, compared to 32% with LHR of 1.12 and 23% with LHR of 1.16 (Fig. 7).

**Fig. 8:**
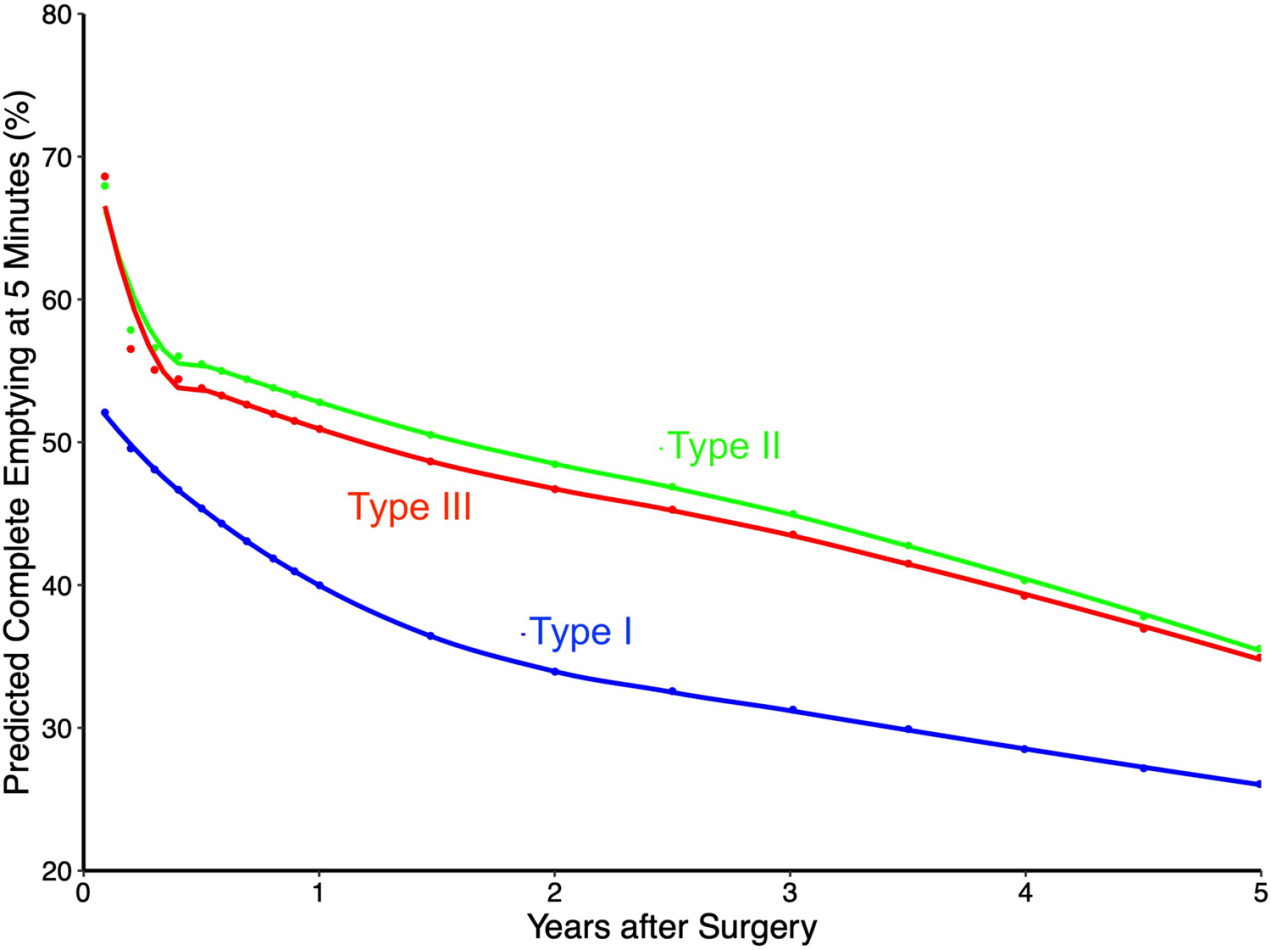
Estimated 5-min complete esophageal emptying according to achalasia manometric subtype

Risked adjusted assessment of complete emptying according to manometric subtype demonstrated decreased complete emptying over time with Type I achalasia, compared to Types II and III achalasia. This corresponded to about 40% complete emptying with Type I achalasia at 1 year, decreasing to 26% by 5 years, compared to 53% and 36% with Type II achalasia, at 1 and 5 years, respectively (Fig. 8).

### Postoperative length-to-height ratio

**Fig. 9:**
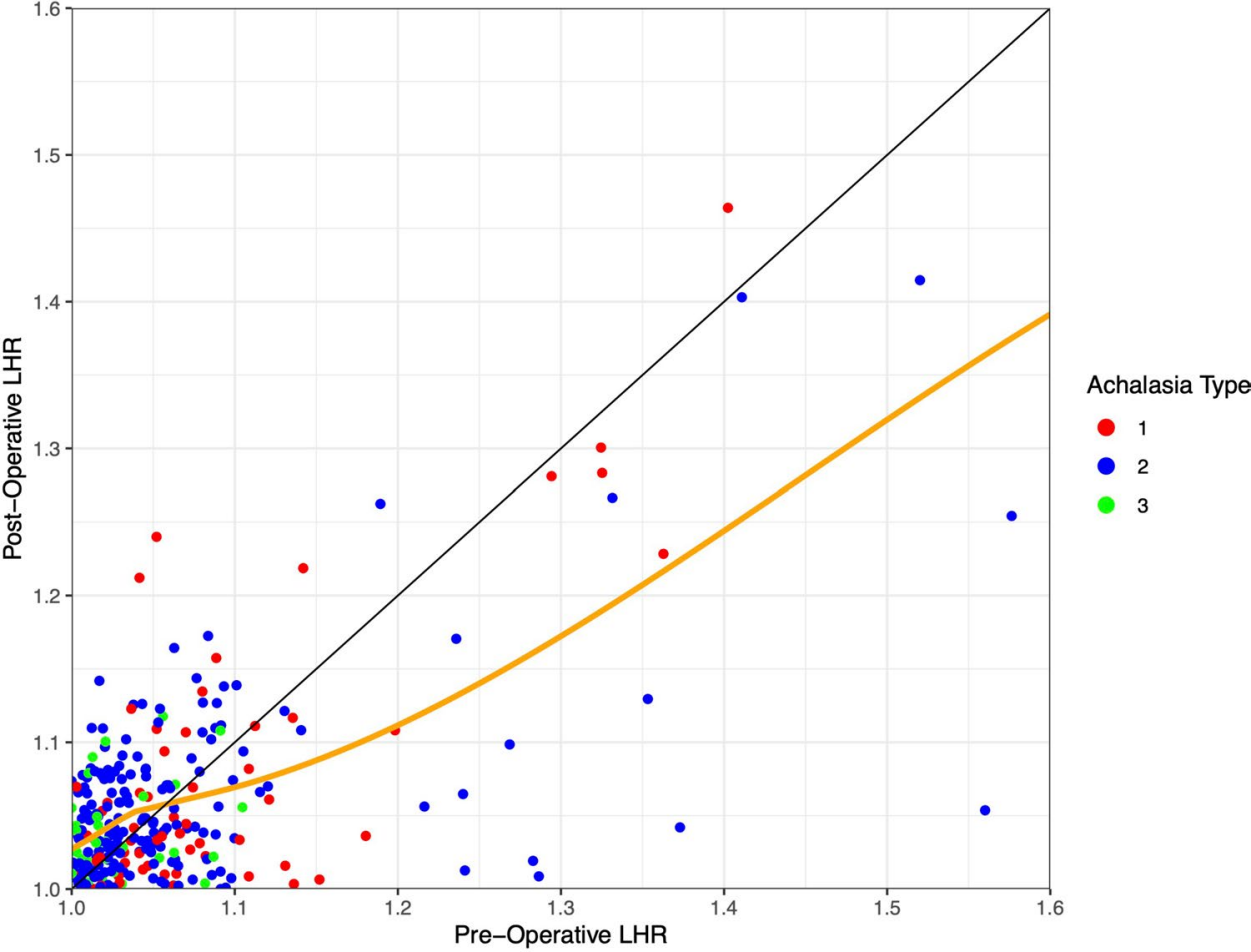
Preoperative vs postoperative length-to-height ratio. Each point represents an individual patient, with color signifying their manometric subtype. The black diagonal line represents a slope of 1, with values to the right of the line indicating a decrease in LHR following myotomy. The orange line represents a Loess curve. *LHR* length-to-height ratio

Postoperative LHR demonstrated minimal change in tortuosity when initial LHR was <1.06. Conversely, when initial LHR was ≥1.06, LHR tended to decrease following myotomy, signifying reduced tortuosity (Fig. 9).

## Discussion

### Principle findings

Preoperative esophageal width >3 cm and age >68 years were the characteristics most predictive of increased preoperative esophageal tortuosity. After adjusting for baseline patient characteristics, including manometric subtype, increasing preoperative LHR, signifying increasing tortuosity, was the only characteristic highly predictive of both symptom relief and complete emptying following myotomy. Manometric subtype, on the other hand, was predictive of only complete emptying. Lastly, severity of tortuosity tends to improve following myotomy when the esophagus is initially tortuous.

### Preoperative characteristics and LHR

Most patients with achalasia present with a relatively straight esophagus, with increased preoperative age and esophageal width associated with increased tortuosity at presentation. It is possible that age may be a surrogate for duration of disease, as the esophagus may become more torturous with ongoing obstruction over time [15]. The association of increased width with increased tortuosity is to be expected, as progression to “end-stage” achalasia, in which the esophagus is both dilated and tortuous, is well-known [16, 17]. Interestingly, manometric subtype was minimally predictive of the severity of tortuosity, suggesting that although we often associate a dilated torturous esophagus with Type I achalasia, tortuosity is a separate phenomenon.

### Symptom relief

Length-to-height ratio was highly predictive of incomplete postoperative symptom relief compared to other preoperative variables, including manometric subtype, even years following myotomy. In contrast to previous work, which has typically categorized tortuosity into “sigmoid” vs “non-sigmoid” groups, we examined tortuosity as a continuous variable, clearly demonstrating that deviation from an LHR of 1.0 is associated with a subtle but progressive deterioration in symptom relief [3, 18, 19]. Previous literature suggests that myotomy is highly effective in relief of symptoms, with 80–93% of patients experiencing adequate palliation 2–5 years postoperatively; as a result, the observed differences in symptom relief of about 4% are substantial, especially since our analysis adjusts for other baseline patient characteristics, including manometric subtype [20, 21].

Previous studies have identified differences in outcomes according to achalasia manometric subtype, with patients with Type III achalasia faring the worst and Type II the best [22–24]. We observed a similar pattern, which emerged after about three years; however, with our estimates controlling for esophageal morphology, which previous studies did not, the incremental contribution of subtype on symptom relief was subtle and much less than 15–30% as previously described [22]. This raises the question whether differences in outcomes should truly be attributed solely to manometric subtype/pattern. For years, the Japan Esophageal Society has incorporated assessment of esophageal morphology into their classification of achalasia [17]. Although LHR appears to predict outcomes more effectively than previous assessments of the angle-based Japanese classification suggest, the Japan Esophageal Society was clearly on the right track – future iterations of achalasia classification should incorporate assessment of esophageal morphology [19, 25, 26].

### Complete esophageal emptying

Length-to-height ratio, preoperative esophageal width, and manometric subtype were the variables most predictive of longitudinal postoperative complete esophageal emptying. Increasing LHR was associated with a gradual decline in estimated complete emptying, with a more precipitous decrease with LHR greater than about 1.09. The decrease in complete emptying with increased tortuosity was more pronounced than that observed with symptom relief, corroborating our previous finding that esophageal emptying may be dissociated from symptom relief [27].

Although we are not aware of any prior assessments of esophageal emptying based on tortuosity, width as a risk factor is supported by Tsuboi and colleagues, who found esophageal width to be associated with slower esophageal clearance [28]. The effect of manometric subtype on complete emptying was explained largely by the Type I subtype, which emptied much less frequently than Types II/III, perhaps as expected given its lack of panesophageal pressurization [29].

### Postoperative tortuosity

When baseline length-to-height ratio was ≥1.06, postoperative tortuosity tended to improve following myotomy, suggesting that some tortuosity results from distal obstruction and that morphologic changes in achalasia are not always permanent. Our findings are supported by Maruyama et al., who noted improvement in the severity of esophageal angulation following POEM for sigmoid esophagus [30]. Similarly, Salvador and colleagues subjectively described less tortuosity following laparoscopic Heller myotomy with “pull-down” technique in patients with sigmoid esophagus [18]. Overall, this provides one explanation as to why patients with severe morphologic changes can benefit from myotomy and may not require esophagectomy.

Because LHR is quantitative, its pre- and postoperative measurement provides a novel straightforward way to grade response to therapy, using nothing more than measurements from the already ubiquitous barium esophagram. Although it remains to be seen whether postoperative improvement in LHR, or lack thereof, prognosticates response to myotomy, its assessment carries promise as a potential objective endpoint to supplement subjective symptom assessment.

### Strengths and limitations

While previous studies have focused on small cohorts with severe morphologic changes, our use of the length-to-height ratio sheds new light on the effect of tortuosity as an objective continuum, rather than at its extremes. Given that the choice of myotomy technique is often dictated by our treatment algorithm, we included patients that underwent both Heller myotomy and POEM, allowing us to examine patients with characteristics spanning the full gamut of achalasia pathology. As an example, if we had only used Heller myotomy, we would not have been able to assess Type III achalasia, as such patients typically undergo POEM. As a result, our findings are generalizable to all patients with achalasia and are representative of outcomes following optimal contemporary treatment based on preoperative patient characteristics. Lastly, we utilized contemporary random forest machine learning methods to generate risk-adjusted estimates, a step that has not been undertaken previously.

There are several limitations, the first being we did not examine postoperative re-intervention, an important endpoint warranting future attention. Although five-year estimates in a large cohort are encouraging, achalasia is a chronic disease, and long-term outcomes based on tortuosity have yet to be determined.

Additionally, tortuosity would ideally be measured in three dimensions, a limitation of the barium esophagram. That said, the anatomic position of the esophagus, with the spine posterior, likely results in development of tortuosity in the coronal plane, which is captured on TBE. In a similar vein, increased age may have been predictive of increased LHR in part due to age-related postural changes (i.e., kyphosis), which could result in smaller height measurements and therefore larger LHR. Inconsistent patient positioning during radiographs might cause similar problems with measurement, although the TBEs were performed according to a prespecified protocol that specifies patients be in an upright position.

Due to the infrequency of extreme tortuosity, the majority of length-to-height ratios fell within a small range, such that we were unable to reliably estimate symptom relief for patients with exceedingly large LHRs. That said, it’s these patients, with LHR of approximately 1.2 or greater, that experts consistently view as “sigmoidal” and whom we already know fare poorly [3, 5]. More widespread use of LHR will facilitate more precise understanding of outcomes for those on the most severe end of the tortuosity spectrum.

Finally, our findings are only as good as our quality of follow up and variables measured. That said, our institution is a high-volume center with a robust systematic approach to postoperative achalasia follow up; further studies with comparable cohorts may be few and far between.

## Conclusion

Increased esophageal tortuosity, as approximated by length-to-height ratio, was the only preoperative variable highly predictive of both worsened longitudinal postoperative symptom relief and incomplete esophageal emptying. Despite relatively large changes in esophageal emptying according to LHR, changes in symptom relief were more subtle, highlighting the discordance between objective and subjective patient evaluation. Following adjustment for baseline patient characteristics, including morphology, postoperative symptom relief varied minimally according to achalasia manometric subtype, questioning whether the large subtype-based differences in outcomes previously reported should be attributed entirely to manometric pattern. Overall, these findings highlight the importance of esophageal morphology in achalasia outcomes and support its inclusion in future iterations of achalasia classification.

## Supplementary Information

Below is the link to the electronic supplementary material.

Supplementary file1 (DOCX 30 KB)

Supplementary file2 (DOCX 15 KB)

Supplementary file3 (TIFF 21096 KB)

Supplementary file4 (JPG 21096 KB)
