# Supplementary material for: Esophageal tortuosity in achalasia: increased length-to-height ratio predicts inferior symptom relief and esophageal emptying following myotomy
Source: Surg Endosc. 2024 Oct 14;39(1):480–91. doi: 10.1007/s00464-024-11200-3 (PMC11666731; doi:10.1007/s00464-024-11200-3)
Supplement: Supplementary file 1 — Supplementary file1 (DOCX 30 KB) [file 464_2024_11200_MOESM1_ESM.docx]

**Appendix 1: Random Forest Supplemental Material**

**Random Forest Methodology**

The following is a description of the process used to assess the relationship of preoperative patient characteristics with preoperative length to height ratio (LHR). Assessment of post-operative longitudinal symptom relief and esophageal emptying relied on similar concepts, with use of boostmtree to account for the longitudinal nature of the endpoints of interest (symptom relief and esophageal complete emptying) [1].

***Motivation***

Traditional parametric modeling of the hazard function and risk factors modulating the risk for preoperative LHR require assumptions about the underlying process and assumes an additive model, with possibly a few first-order interactions explored. We have selected a completely nonparametric method for this analysis to avoid model assumptions and maximize sensitivity to interactions among variables and linear or nonlinear relationships to LHR.

***Method***

Overview

Random Forests (RF) is a nonparametric statistical method that makes no distributional or functional assumptions about covariate relationships to the response [2]. RF is a robust, nonlinear technique that optimizes predictive accuracy by fitting an ensemble of trees to stabilize model estimates.

RF analysis is not parsimonious but uses all variables available in the construction of a response predictor. The RF variable selection process uses the variable importance measure (VIMP), along with minimal depth, a property derived from the construction of each tree within the forest, to assess the effect of variables on forest prediction [2–4].

All variables considered (*p*) for the analysis are used to grow each tree. However, to overcome the problem in classic variable selection procedures of the order in which variables are selected (e.g., by any forward selection process), at each node split, the ceiling of the square root of *p* candidate variables is selected at random from among the *p* variables and the log-rank test used to optimize splitting among only these candidate variables. In this case, 21 covariables (*p*) were explored in building each tree using the RF-SRC R package, and 11 candidate variables were selected for each split [3, 4]. A forest of 5000 trees was grown.

VIMP

Variable importance was originally defined in classification and regression tree analyses (CART) using a measure involving surrogate variables [5]. The most popular VIMP method uses a prediction error approach based on 2 concepts: The first is that on average, bootstrap sampling leaves out 36.8% of observations. These observations, known as Out of Bag (OOB) samples, can be sent up the tree to provide predictions of their outcomes. The second concept involves “noising-up” variables by randomly permuting their values, which provides a variable with identical statistical properties but connected to random individuals in the sample. VIMP for a variable $x_{v}$ is the difference between prediction error based on OOB results when $x_{v}$ is noised up compared with prediction error under the observed values [2, 6–8].

Because VIMP is the difference between OOB prediction error before and after permutation, a large VIMP value indicates that variable misspecification detracts from the predictive accuracy in the forest, VIMP close to 0 indicates the variable contributes nothing to predictive accuracy, and negative values indicate the predictive accuracy improves when the variable is misspecified. In the latter case, we assume noise is more informative than the true variable. As such, we may wish to ignore variables with negative and near 0 values of VIMP, relying on large positive values to indicate that the predictive power of the forest is dependent on those variables.

***Visualization of Random Forest Analysis***

Overview

Once we identify variables to investigate, we use dependence plots to understand the shape and direction of how a variable is related to the response [9, 10]. Variable dependence plots give us an idea of the overall trend of a variable-response relation based on risk-adjusted curves for each observation, and partial dependence plots indicate the completely risk-adjusted relation. The 2 plots yield generally similar results, although variable dependence is more computer-intensive. To graphically investigate variable interactions within the forest model, we generate variable dependence and partial dependence conditioning plots (coplots) [11, 12].

Variable Dependence

Variable dependence plots show the predicted response as a function of a covariate of interest, where each observation is represented by a point on the plot of each device’s predicted time-related curve. Each point represents an individual observation, where the predicted response is dependent on the full combination of all other covariates, not only on the covariate of interest. Interpretation of variable dependence plots can give shape and direction of the response in general terms, as point predictions are a function of all covariates in that particular observation. Variable dependence is straightforward to calculate, requiring only the predicted response for each observation.

Partial Dependence

Partial variable dependence plots are an alternative to variable dependence. Partial plots are generated by integrating out the effects of all variables beside the covariate of interest. Partial dependence data are constructed by selecting points evenly spaced along the distribution of the $X$ variable of interest. For each value ($X=x$), we calculate the average RF prediction over all other covariates in $X$ by

$$\tilde{f}(x)=\frac{1}{n}\sum_{i=1}^{n} \hat{f}(x,x_{i,o}),$$

where $\hat{f}$ is the predicted response from the random forest and $x_{i,o}$ is the value for all covariates other than $X=x$ for the observation $i$ [9]. Essentially, we average a set of predictions for each observation in the training set at the value of $X=x$. We repeat the process for a sequence of $X=x$ values to generate the estimated points and create a partial dependence plot.

***Missing Value Imputation***

There are 2 modeling issues when dealing with missing data values: How does the algorithm build a model when values are missing from the training data and how does the algorithm predict a response when values are missing from the test data? The standard procedure for linear models is either to remove or impute the missing data values before modeling; that is, removing the missingness is done by either removing observations with missing data (row-wise) or removing the variable with missing values (column-wise). Removal is a simple solution but may bias results when either observations or variables are scarce.

Rather than impute missing values before growing the forest, RF uses adaptive forest imputation, a method that imputes missing values “just in time” [13]. At each node split, the set of candidate variables is checked for missing values. Missing values are then imputed by randomly drawing values from non-missing data within the node. The split-statistic is then calculated on observations that were not missing values. The imputed values are used to sort observations into the subsequent daughter nodes, then discarded before the next split occurs. The process is repeated until the stopping criteria are reached and all observations are sorted into terminal nodes.

Adaptive tree imputation still assumes values are missing at random [14]. At each imputation step, the random forest assumes that similar observations are grouped together within each node. The random draws used to fill in missing data do not bias the split rule, but sort observations similar in non-missing data into like nodes. An additional feature of this approach is the ability of predicting on test set observations with missing values.

**REFERENCES**

1. Breiman L: Random forests. Machine Learning 2001;45:5-32.

2. Ishwaran H, Kogalur UB, Blackstone EH, Lauer MS: Random survival forests. Ann Appl Stat 2008;2:841-60.

4. Ishwaran H, Kogalur UB: Consistency of Random Survival Forests. Stat Probab Lett 2010;80:1056-64.

5. Ishwaran H, Kogalur UB, Chen X, Minn AJ: Random Survival Forests for High-Dimensional Data. Statist Anal Data Mining 2011:115-32.

6. Ishwaran H, Kogalur UB: Random forests for survival, regression, and classification (RF-SRC), R package version 1.6.0, URL: <http://cran.r-project.org/web/packages/randomForestSRC/index.html>. 2015.

7. R Core Team. R: A language and environment for statistical computing. R: Foundation for Statistical Computing. <http://www.R-project.org>. 2013

8. Breiman L, Friedman JH, Olshen R, Stone C: Classification and regression trees. Monterey, CA: Wadsworth and Brooks; 1984.

9. Liaw A, Wiener M: Classification and regression by randomForest Rnews 2002;2/3:18-22.

10. Ishwaran H: Variable importance in binary regression trees and forests. Electron J Statist 2007;1:519-37.

11. Ishwaran H, Kogalur UB, Gorodeski EZ, Minn AJ, Lauer MS: High-dimensional variable selection for survival data. J Am Stat Assoc 2010;105:205-17.

12. Friedman JH: Greedy function approximation: a gradient boosting machine. Ann Statist 2000;29:1189-232.

13. Ehrlinger J: ggRandomForests: visually exploring Random Forests. R package version 1.1.4. URL: <http://cran.r-project.org/package=ggRandomForests>. 2015.

14. Chambers JM, Hastie TJ: Statistical models in S: Wadsworth & Brooks/Cole; 1992.

15. Cleveland WS: Visualizing data: Summit Press; 1993.

16. Rubin DB: Inference and missing data. Biometrika 1976;63:581-92.
