# Supplementary material for: Esophageal tortuosity in achalasia: increased length-to-height ratio predicts inferior symptom relief and esophageal emptying following myotomy
Source: Surg Endosc. 2024 Oct 14;39(1):480–91. doi: 10.1007/s00464-024-11200-3 (PMC11666731; doi:10.1007/s00464-024-11200-3)
Supplement: Supplementary file 2 — Supplementary file2 (DOCX 15 KB) [file 464_2024_11200_MOESM2_ESM.docx]

**Supplemental Table 1:** Preoperative predictor variables included in the random forest models for analysis. ASA, American Society of Anesthesiologists; TBE, timed barium esophagram

| **Variables** | |
| --- | --- |
| Age  Sex  Race  Body mass index  Total Eckardt score  Eckardt dysphagia severity  Eckardt regurgitation severity  Eckardt chest pain severity  Eckardt weight loss  Diverticulum  Hiatal hernia | Integrated relaxation pressure  ASA class  Achalasia manometric subtype  Previous botulinum toxin injection  Previous pneumatic dilation  Previous simple dilation  Barium volume ingested for TBE  TBE height and width at 1 and 5 minutes  Length-to-height ratio  Surgical approach^a^ |
| ^a^Per-oral endoscopic myotomy, laparoscopic Heller myotomy, robotic Heller myotomy, or open Heller myotomy | |
